# Left Internal Mammary Artery Injury Requiring Resuscitative Thoracotomy: A Case Presentation and Review of the Literature

**DOI:** 10.1155/2012/459841

**Published:** 2012-12-04

**Authors:** Ammar Al Hassani, Yassir Abdul Rahman, Ahad Kanbar, Ayman El-Menyar, Abubaker Al-Aieb, Mohammad Asim, Rifat Latifi

**Affiliations:** ^1^Section of Trauma, Department of Surgery, Hamad General Hospital (HGH), Hamad Medical Corporation, P.O. Box 3050, Doha, Qatar; ^2^Weill Cornell Medical School, P.O. Box 24144, Doha, Qatar; ^3^Clinical Research, Section of Trauma, Department of Surgery, HGH, Hamad Medical Corporation, Doha, Qatar; ^4^Department of Surgery, University of Arizona, Tucson, AZ 85724, USA

## Abstract

*Background*. Penetrating injuries to the chest and in particular to the heart that results in pericardial tamponade and cardiac arrest requires immediate resuscitative thoracotomy as the only lifesaving technique and should be performed without delay. *Objective*. To describe an external cardiac tamponade caused by massive tension hemothorax from penetrating injury of the left internal mammary artery (LIMA). *Method*. A case presentation treated at the Level I trauma center at Hamad General Hospital, in Doha, Qatar and review of the literature on LIMA injuries reported cases. *Results*. LIMA injury as a cause of hemothorax is not uncommon, but to our knowledge our case is the first massive tension hemothorax with witnessed cardiac arrest reported in the literature requiring emergency thoracotomy, performed in trauma room, with full recovery. *Conclusion*. Injury to the LIMA with massive tension hemothorax requires immediate resuscitative thoracotomy.

## 1. Background

The concept of a thoracotomy as a resuscitative measure began with Schiff's promulgation of open cardiac massage in 1874 [1]. The value of resuscitative thoracotomy (RT) in resuscitation of the patient with penetrating injuries to the heart with witnessed loss of vital signs has been demonstrated [2]. Overall analysis of the available literature indicates that the success of RT approximates 35% in penetrating cardiac wound, patient arriving in shock, and 15% for all penetrating wounds. Conversely, patient outcome is relatively poor when RT is done for blunt trauma, 2% survival in patients in shock and less than 1% survival with no vital signs [3]. Current indications for RT are penetrating injury to the chest and “cardiac box” with witnessed loss of vital signs and persistent, severe hemorrhagic shock that precludes transport to the OR. Direct injury to the heart resulting in pericardial tamponade and cardiac arrest are most common. To our knowledge, this is the second case of left internal mammary artery (LIMA) injury causing massive tension hemothorax, not relieved by a chest tube, reported in the literature.

## 2. Case Presentation 

A 32-year-old male sustained multiple stab wounds to the left chest, approximately 45 minutes before he was brought to the trauma room. Less than five minutes after arriving to the trauma resuscitation room (TRU) the patient had no detectable pulse and blood pressure. A left chest thoracotomy tube was inserted and approximately 1800 mL of blood was immediately evacuated, but the patient's vital signs were not detectable. Left resuscitative thoracotomy was done and large amount of blood causing massive tension hemothorax and external pericardial tamponade was evacuated. With simultaneous release of tension hemothorax and resuscitation with blood transfusion, the cardiac activity returned with palpable pulses. Left chest was packed and patient was taken to the operating room.

Left thoracotomy exploration revealed transection of the left mammary artery at the third intercostal space. Combining direct approach to LIMA, by extending the stab wound at the third intercostal space and lifting the chest wall in order to access the distal segment of the LIMA, ligation of both ends was achieved. Further exploration revealed injury to the left diaphragm from another separate stab wound. Exploratory laparotomy revealed approximately 4-5 cm laceration to the anterior stomach with large food spillage in the peritoneal cavity. Following closure of thoracotomy with two chest tubes placement and closure of the abdomen, patient was transferred to intensive care unit for further resuscitation, hemodynamically stable. He was extubated after operative day 3 and discharged to home seven days later, having recovered fully. 

## 3. Discussion 

Good functional recovery and outcome, as in our paper, from tension massive hemothorax with prehospital arrest that resulted from penetrating internal mammary injury, requiring RT in the TRU has not been reported. There are few reports of internal mammary injury that have resulted in pericardial tamponade and hemothorax three after penetrating trauma, and one with blunt trauma; reported previously (Table 1) [4–7].

**Figure 1:**
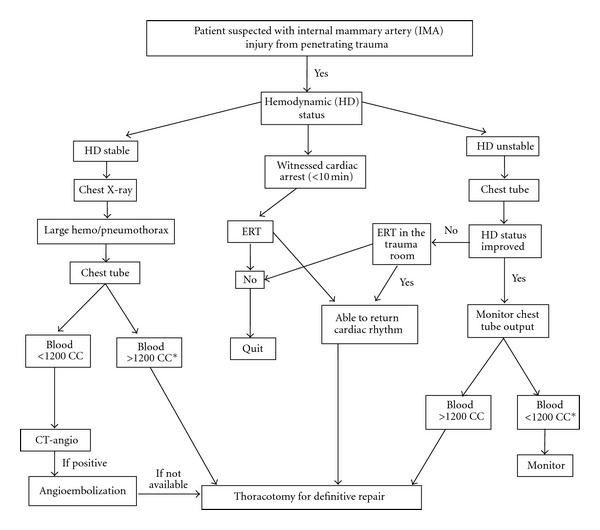
Algorithm approach for suspected internal mammary artery (IMA) injury. Although current resuscitation by thoracotomy is bleed >1500 CC, we believe that if >1200 CC the patient should undergo thoracotomy. IMA: Internal mammary artery, ERT: Emergency resuscitative thoracotomy (courtesy from Rifat Latifi).

Injury to Internal mammary artery is infrequently reported in literature. It can be a result of penetrating or blunt trauma, both of rare in occurrence but still with serious consequences [8, 9]. Others have described internal mammary injury that had occurred from central line insertion [10]. The presentation of internal mammary injury varies from relatively stable patient that can be studied in a timely manner to identify the source of hemothorax, to a delayed presentation of massive hemothorax that mandate thoracotomy [11, 12]. Many treatment options have been described so far, but all depends on the hemodynamic status of the injured patients. If a patient is not in cardiac arrest, release of hemothorax with a chest tube is the first line of intervention. While there is not enough experience reported with this injury, chest tube insertion will release tension hemothorax. If on the other hand placement of chest tube is not effective enough or if patient presents with witnessed cardiac arrest of less than 10 minutes, then ERT should be performed, if there is a surgeon in the institution rendering the care. The injured internal mammary may be treated based on the clinical presentation. This management includes angioembolization or open surgery and ligation of the injured vessel [13]. We have proposed an algorithm based on current treatment approaches to penetrating chest trauma (Figure 1). 

## 4. Conclusion

 This paper demonstrates that injury to the left internal mammary artery with massive tension hemothorax requires immediate resuscitative thoracotomy and should be performed immediately. 

## Figures and Tables

**Table 1:** Review of literature of internal mammary artery injuries.

Study	Mechanism of injury	Hemodynamic	Pericardial tamponade	Massive hemothorax	Surgery	Outcome
Curley et al. (1987) [4]	Penetrating	Unstable	Early	Early	Sternotomy	Alive
Vinces (2005) [5]	Penetrating	Stable	Delayed	Delayed	Thoracotomy	Alive
Holt et al. (2005) [6]	Penetrating	Stable	Delayed	No	Sternotomy	Alive
Irgau et al. (1995) [7]	Blunt	Stable	Delayed external tamponade	No	Sternotomy	Alive
Current case	Penetrating	Unstable	Early external tamponade	Early	Thoracotomy	Alive

## Abbreviations

LIMA: Left internal mammary artery
RT: Resuscitative thoracotomy.
